# Modulation of Antioxidant Enzymatic Activities by Certain Antiepileptic Drugs (Valproic Acid, Oxcarbazepine, and Topiramate): Evidence in Humans and Experimental Models

**DOI:** 10.1155/2013/598493

**Published:** 2013-12-17

**Authors:** Noemí Cárdenas-Rodríguez, Elvia Coballase-Urrutia, Liliana Rivera-Espinosa, Arantxa Romero-Toledo, Aristides III Sampieri, Daniel Ortega-Cuellar, Hortencia Montesinos-Correa, Esaú Floriano-Sánchez, Liliana Carmona-Aparicio

**Affiliations:** ^1^Laboratory of Neurochemistry, National Institute of Pediatrics, 04530 DF, Mexico; ^2^Laboratory of Pharmacology, National Institute of Pediatrics, 04530 DF, Mexico; ^3^Laboratory of Molecular Biology and Genomics, Faculty of Sciences, University City, UNAM, 04150 DF, Mexico; ^4^Laboratory of Experimental Nutrition, National Institute of Pediatrics, 04530 DF, Mexico; ^5^Service of Endocrinology, National Institute of Pediatrics, 04530 DF, Mexico; ^6^Section of Research and Graduate Studies, IPN, 11430 DF, Mexico

## Abstract

It is estimated that at least 100 million people worldwide will suffer from epilepsy at some point in their lives. This neurological disorder induces brain death due to the excessive liberation of glutamate, which activates the postsynaptic N-methyl-D-aspartic acid (NMDA) receptors, which in turn cause the reuptake of intracellular calcium (excitotoxicity). This excitotoxicity elicits a series of events leading to nitric oxide synthase (NOS) activation and the generation of reactive oxygen species (ROS). Several studies in experimental models and in humans have demonstrated that certain antiepileptic drugs (AEDs) exhibit antioxidant effects by modulating the activity of various enzymes associated with this type of stress. Considering the above-mentioned data, we aimed to compile evidence elucidating how AEDs such as valproic acid (VPA), oxcarbazepine (OXC), and topiramate (TPM) modulate oxidative stress.

## 1. Introduction

Neurological diseases are a major cause of health concerns at different life stages and lead to considerable utilization of medical resources [1]. Epilepsy is one of the most common neurological disorders in both children and adults [2, 3]. The term epilepsy describes a group of disorders characterized by the presence of chronic, recurrent, and paroxysmal alterations of the motor and sensory neurological functions secondary to a disorder in the electrical activity of a neuron population [4]. The term epileptic syndrome refers to various disorders characterized by a group of signs and symptoms that occur simultaneously. These signs include the type of crisis, causes, anatomic aspects, precipitating factors, age of onset, severity, prognostics, chronicity, and electroencephalographic activity, and the clinical characteristics are identified based on the patient's age [2, 5].

Epileptic seizures and syndromes are classified according to the International League Against Epilepsy (ILAE), using genetic studies and electroclinical, neuropsychological, and neuroimaging research. Epilepsy can be divided, based on its etiology, into idiopathic disease or disease associated with a hereditary predisposition, as symptomatic or associated with any event that damages the brain, and as cryptogenic or of unknown cause [6, 7].

Currently, the epilepsy prevalence is reported to be five to 10 cases per 1,000 individuals. It is estimated that at least 100 million people worldwide will present with epilepsy at a certain life stage [4, 8]. The ILAE reports that the disease prevalence lies between four and 10 cases per 1,000 individuals, and the incidence lies between 20 and 70 cases per 100,000 individuals per year. The prevalence rate in Latin-American countries is the highest, in the range of 14 to 57 per 1,000 individuals [6, 7].

Epilepsy control using antiepileptic drugs (AEDs) depends on several factors: efficacy, side effects of the hormonal alteration, teratogenicity, pharmacokinetics, interactions between AEDs or other drugs, serum levels, cost, and the neurologist's experience with AED use [9]. The patient may respond in three different manners: remitting seizures spontaneously (without AED use), responding adequately to AED administration, or presenting refractoriness to the treatment drug. The most commonly used AEDs are valproic acid (VPA), oxcarbazepine (OXC), and topiramate (TPM), which are considered the first-option treatments for the diverse manifestations of this pathology.

A wide variety of AEDs have been divided into generations according to their date of introduction to clinical use. These agents are categorized as first- (1857–1978), second- (1993–2009), and third- (2009 to date) generation AEDs. The second- and third-generation drugs are described in Table 1 [10, 11].

## 2. Overview of Valproic Acid, Oxcarbazepine, and Topiramate

**Figure 1:**
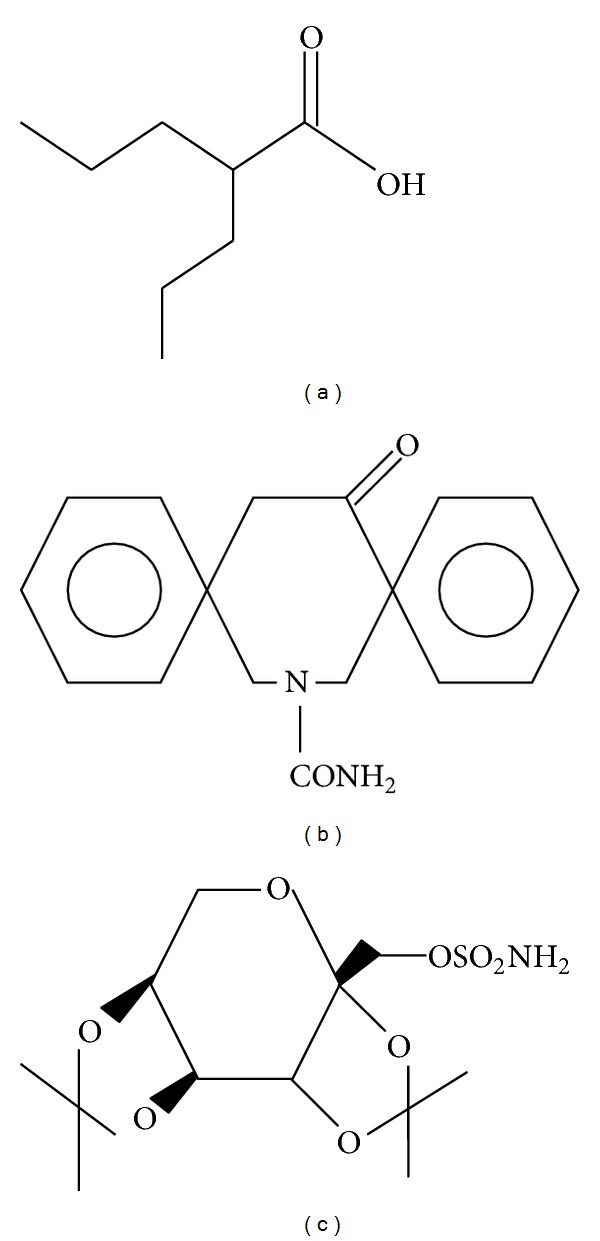
Chemical structures of (a) VPA, (b) OXC, and (c) TPM.

VPA is a carboxylic acid composed of eight carbons and is used to treat several types of epilepsy due to its broad action spectrum and efficiency [12] (Figure 1(a)). The mechanism of action, similarly to that of other AEDs, is not fully known; however, it has been reviewed in various articles. These reports can be divided into two groups: studies suggesting that VPA increases gamma aminobutyric acid (GABA) transmission and research indicating that this AED may directly interact with the neuronal membrane. Löscher [12] studied VPA interference with GABAergic transmission in 1993. This report is based on the observation that VPA increases the levels of the inhibitory neurotransmitter GABA [12]. Other researchers have confirmed Löscher's studies [13–15]. This effect can be produced either by glutamate decarboxylase activation [16, 17]; by the inhibition of GABA-degrading enzymes such as GABA aminotransferase [17], succinic semialdehyde dehydrogenase [18], aldehyde reductase [19], and *α*-ketoglutarate dehydrogenase [17]; or by an increase in glutaminase activity [20]. Alternative mechanisms involve potentiation of the postsynaptic response of GABA, GABA_A_ receptor modulation, and depolarization induced by N-methyl-D-aspartic acid (NMDA) [21–23].

With respect to the mechanism of VPA interactions with the neuronal membrane, it has been reported that this AED decreases the excitatory synaptic potential necessary for the synchronization network and the neuronal firing in the substantia nigra [24–26]. VPA also activates conductance in the potassium channels and interferes with other biochemical pathways related to energy metabolism in the brain. However, to date, this activity has not been proven to be an additional mechanism of AED action [27].

**Figure 2:**
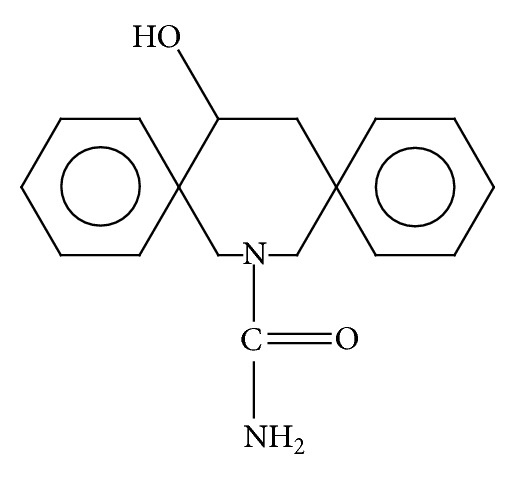
Chemical structure of 10,11-dihydro-10-hydroxy-carbazepine, main active metabolite of OXC.

OXC was developed as a carbamazepine analog (Figure 1(b)) [28], and its pharmaceutical activity occurs mainly through its active metabolite, 10,11-dihydro-10-hydroxy-carbazepine (Figure 2). The mechanisms of action have not been clarified, but it is reported that the principal mechanism involves a blockade of voltage-dependent sodium [29]. *In vitro* electrophysiological and animal studies have demonstrated that this AED activity is based on the interference with transmembranal sodium, calcium, and potassium (i.e., voltage-dependent) ionic currents. These agents also modify the release of certain neurotransmitters, such as glutamate [29, 30].

TPM is a monosaccharide substituted with sulfate groups [2,3:4,5-bis-*O*-(1-methylethylidene)-beta-D-fructopyranose sulfamate] (Figure 1(c)). Five mechanisms that reportedly contribute to its antiepileptic action are as follows: blockage of the sodium channels, which reduces the duration and frequency of the action potentials [31]; a positive the GABA_A_ receptors [32, 33]; inhibition of the ionotropic glutamate receptors alpha-amino-3-hydroxy-5-methyl-4-isoxazolepropionic acid receptor (AMPA)/kainate; a negative modulatory effect of the calcium channels activated by L-type voltage; and inhibition of carbonic anhydrase subtypes II and IV [32].

## 3. Oxidative Stress and Its Role in Epilepsy

In the last two decades, the study of ROS and reactive nitrogen species (RNS) has sparked great interest in clinical and experimental medicine. Both species are (a) generated during the irradiation of ultraviolet (UV) light, X-rays, and gamma rays, (b) products of reactions catalyzed by metals, (c) present in air pollutants; (d) produced by neutrophils and macrophages during inflammation, and (e) byproducts of reactions catalyzed by the electron carriers in the mitochondria [34].

ROS and RNS are known for their dual role in biological systems, as they can be beneficial or harmful. The beneficial effects of ROS can be observed in their physiological role in numerous cellular responses and cell signaling systems. By contrast, at high concentrations, ROS can be important mediators of cell damage to various structures such as lipids, proteins, and nucleic acids. The beneficial effects of ROS are supplemented by the action of nonenzymatic antioxidants and by the antioxidant enzyme system. Despite the presence of the antioxidant defense system to combat oxidative damage caused by ROS, this damage accumulates throughout life [34].

The imbalance between the ROS and RNS levels (such as superoxide, radical O_2_^∙−^; hydrogen peroxide, H_2_O_2_; hydroxyl radical, HO^*∙*^; and nitric oxide, NO^∙^) and the cellular antioxidant defense system (superoxide dismutase, SOD; catalase, CAT; glutathione peroxidase, GPx; glutathione reductase, GR; and glutathione-S-transferase, GST) is defined as “oxidative stress” [34]. Because this disequilibrium can appear at the cellular level (involving the mitochondria, cytochrome P450 system, peroxisomes, and activation of inflammatory cells [35]), it is involved in the development of several diseases such as cancer, atherosclerosis, and arthritis and in neurodegenerative disorders such as epilepsy [34, 36].

The participation of oxidative stress in diseases of the central nervous system (CNS) is well established [37, 38]. The brain is highly sensitive to oxidative damage because this organ contains a large number of easily oxidized fatty acids (20 : 4 and 20 : 6) and a limited antioxidant system [37].

Oxidative stress is strongly implicated during seizures induced by excitotoxicity, due to mitochondrial ROS generation. Since the beginning of the 1990s, oxidative stress has been associated with neuronal hyperexcitation caused by CNS diseases [39]. Dalton, in 1995, was the first to identify brain damage induced by the presence of oxidative stress in an animal experimental model [40].

The presence of NO^∙^ is known to be a cause of seizures [41]. NO^∙^ is formed from high concentrations of inducible nitric oxide synthase (iNOS). The role of oxidative stress in pentylenetetrazole-induced epilepsy has been proven in rodents [42–44]. The increased activity of glutamatergic systems induces status epilepticus and causes an energy imbalance, increasing ROS formation [45]. Several studies have linked seizures and cell damage to the excitotoxicity induced by pentylenetetrazole [46]. Seizures are linked to the increased release of glutamate and NMDA receptor activation. In fact, during epileptic seizures induced by different models, there is an extracellular Ca^2+^ concentration decrease and a cytosolic Ca^2+^ concentration increase [47].

The effects mediated by Ca^2+^ during excessive glutamate receptor activation (excitotoxicity) lead to neuronal degeneration and give rise to oxidative stress. The phospholipase A_2_-dependent activity of Ca^2+^ mediated by glutamatergic receptors liberates arachidonic acid (AA), which generates O_2_^∙−^  through its metabolism by lipoxygenases and cyclooxygenases for eicosanoid formation [48].

The constant formation of NO^∙^ by the glia is neurotoxic because it increases the neuronal sensitivity to this reactive species. The neurotoxic action of NO^∙^ is likely caused by the formation of peroxynitrite (ONOO^−^), which is rapidly formed by the reaction of NO^∙^ with O_2_^∙−^. Under conditions of energy deficit and elevated intracellular Ca^2+^ concentration, xanthine oxidase generates O_2_^∙−^. In this environment, lactic acid is generated, which promotes the release of Fe^2+^, the Haber-Weiss reaction, and the production of HO^∙^ [49]. Furthermore, ONOO^−^ can react with tyrosine in proteins to form 3-nitrotyrosine (3-NT) [50].

## 4. Antioxidant Enzymes Induced by VPA, OXC, and TPM: Evidence in Humans and Experimental Models

It is established that VPA, OXC, and TPM are capable of modulating the oxidant-antioxidant system. Particularly, enzyme antioxidant activity studies have demonstrated that the administration of certain AEDs causes decrease in enzyme activity, increase in the antioxidant system, or even the absence of an effect upon the antioxidant system.

VPA can modulate negatively or positively the enzymatic activity. Chaudhary and Parvez, in 2012, found a significant reduction of GST, GR, GPx, SOD, and CAT activity. They also observed significantly increased xanthine oxidase activity and lipid peroxidation levels in the cerebellum and cerebral cortex of rats [66]. Zhang et al., in 2011, observed a significant decrease in the antioxidant activity of SOD and CAT, a significant increase in the myeloperoxidase (MPO) activity, and increased levels of lipid peroxidation in epileptic children [65]. Varoglu et al., in 2010, observed significantly increased 8-hydroxyguanosine (8-OHG) levels in the serum of epileptic patients [63]; Peker et al., in 2009, found a significant increase of NO^∙^ in the serum of epileptic children [61]. Martínez-Ballesteros et al., in 2004, observed significantly increased lipid peroxidation in epileptic patients treated with VPA [59]. Other studies have revealed a positive regulatory effect on the antioxidant activity of certain proteins [56, 67–69].

A few *in vivo* and *in vitro* studies have reported the effect of the OXC and TPM AEDs on the antioxidant defense system, the lipid peroxidation levels, and the ROS levels. Positive regulation of the antioxidant system by OXC has not been reported to date. Cardile et al. in 2001 and Pavone and Carile, in 2003, demonstrated increased ROS levels in cultured astrocytes, which caused negative regulation [70, 71]. Agarwal et al., in 2011, found significantly increased lipid peroxidation levels and significantly decreased levels of reduced glutathione (GSH) in rats with pentylenetetrazole-induced epilepsy [72].

Cardile et al., in 2001, and Agarwal et al., in 2011, reported a negative regulatory effect of TPM. With respect to the positive regulatory effect of TPM on the antioxidant system, a significant increase in SOD, CAT, GPx, and neuronal nitric oxide synthase (nNOS) activities, as well as in GSH levels, has been observed. Significantly decreased lipid peroxidation levels have also been observed in experimental epilepsy models [43, 73–76]. Our research group has demonstrated for the first time that TPM has direct antioxidant activity *in vitro* against O_2_^∙−^ , H_2_O_2_, HO^*∙*^, and hypochlorous acid (HOCl) in a concentration-dependent manner. In this study, we demonstrated that the scavenging activity of TPM might explain its neuroprotective properties [77]. Human studies have evaluated the effects of AEDs on the antioxidant system; see Table 2.

Although there is evidence in humans and experimental models that these AEDs (VPA, OXC, and TPM) modulate the activity of antioxidant enzymes, this evidence is not conclusive. It is necessary to study further how AEDs induce effects in antioxidant capacities in different types of epilepsy. In particular, our research group is interested in determining the activity of the antioxidant enzymes and their modulation by AEDs in epileptic pediatric patients.

## 5. Therapeutic Relevance

To date, the mechanisms involved in the etiopathogenesis of epilepsy remain unclear; however, the evidence showed that oxidative stress is involved in the epilepsy development. Therefore, the knowledge that current AEDs can modulate other systems opens a new therapeutic window for the population suffering from epilepsy and other chronic and degenerative CNS diseases, in which oxidative stress is one of the mechanisms underlying these pathologies.

## Figures and Tables

**Table 1:** Primary second- and third-generation AEDs (listed in chronological order). Modified from Shorvon (2009) and Löscher and Schmidt (2011) [10, 11].

Second generation	Third generation
Chlordiazepoxide Sulthiame Diazepam Carbamazepine Valproate Clonazepam Clobazam	Vigabatrin
	Zonisamide
	Lamotrigine
	Oxcarbazepine
	Felbamate
	Gabapentin
	Topiramate
	Tiagabine
	Levetiracetam
	Pregabalin
	Rufinamide
	Lacosamide
	Ethyl carbazepine
	Retigabine

**Table 2:** Effect of AEDs on the activity of antioxidant enzymes or oxidative stress markers in epileptic patients.

AEDs	Antioxidant enzymatic activity/oxidative stress markers	Findings	Ref
VPA	GPx	Kurekci et al., in 1995, found a significant increase in GPx activity in children diagnosed with epilepsy.	[51]

VPA and carbamazepine	GSH, GPx, SOD, and malonaldehyde (MDA)	Cengiz et al., in 2000, evaluated the effect of VPA and carbamazepine on the levels of GSH, GPx, SOD, and lipid peroxidation in the erythrocytes of 30 children diagnosed with epilepsy and compared with 25 healthy children. The authors found that during a one-year treatment with VPA (in 16 children) or carbamazepine (in 14 children), the GPx levels were significantly increased, but the GSH levels were significantly decreased. With combined drugs, they were no significant differences in the SOD activity and lipid peroxidation levels.	[52]

VPA and carbamazepine	GPx, SOD, and MDA	Y$\ddot{\text{u}}$ksel et al., in 2001 and 2000, found a significant increase in the levels of lipid peroxidation and decreased GPx activity in the serum of 14 children treated with VPA for two years compared with that found in 27 healthy children. The SOD serum levels increased significantly during the first year. In 13 children diagnosed with epilepsy and then treated with carbamazepine for two years, lipid peroxidation increased significantly in the serum, compared with the control group. During the second year of treatment with carbamazepine, the serum SOD levels were significantly higher, compared with the control group and with the same group before treatment.	[53, 54]

VPA and carbamazepine	Se, GPx, and Cu/Zn-SOD	Verrotti et al., in 2002, found that 36 children with epilepsy and no treatment exhibited no significant differences in the serum levels of Se, GPx, and Cu/Zn-SOD, compared with the control group (14 children). One year after beginning therapy with VPA (in 22 patients) or carbamazepine (in 14 patients), the values of these parameters were unchanged.	[55]

VPA and carbamazepine	SOD, GPx, and MDA	Solowiej and Sobaniec, in 2003, found that 25 children treated with VPA, 16 children treated with carbamazepine, and 27 children treated with polytherapy (carbamazepine + VPA) exhibited a significant decrease in the serum SOD activity, compared with 61 healthy children. The serum GPx activity was significantly increased in all patient groups except in those receiving combination therapy, compared with the control group. The lipid peroxidation levels in the serum were significantly increased in all patients.	[56]

VPA	GPx	Hamed et al., in 2004, found that 14 adult patients without treatment exhibited no significant decrease in GPx activity but exhibited a significant reduction in the total antioxidant capacity in the serum. Fifty-five patients with epilepsy treated using VPA exhibited a significant increase in their serum GPx levels and total antioxidant capacity.	[57]

OXC	GPx, SOD, and MDA	Bolayir et al., in 2004, found that the GPx activity, SOD activity, and lipid peroxidation levels in erythrocytes were significantly different after one year of therapy with oxcarbazepine. This study was performed in 13 adult patients, and the results were compared with the results obtained from 15 healthy adults and from the same patients before monotherapy.	[58]

VPA	MDA	Martínez-Ballesteros et al., in 2004, found a significant increase in lipid peroxidation in 76 patients compared with the control group.	[59]

VPA and Carbamazepine	SOD, GPx, GR, and MDA	Sobaniec et al., in 2006, evaluated the effect of therapy with AEDs and how these drugs changed the SOD, GPx, and GR activity and the lipid peroxidation levels in the erythrocytes of 90 pediatric patients and 61 healthy children. The activity of the antioxidant enzymes was significantly higher. The lipid peroxidation levels were significantly lower in children treated only with carbamazepine. In children treated with VPA, the activity of all antioxidant enzymes was lower. Higher levels of lipid peroxidation were concurrently demonstrated. In patients treated with combination therapy, the SOD activity was lower, whereas the activity of GPx and GR was higher. In addition, lower lipid peroxidation levels were displayed.	[60]

VPA	NO^•^, SOD, CAT, and MDA	Peker et al., in 2009, investigated the effect of VPA on the serum levels of NO^•^, lipid peroxidation, and certain antioxidant enzymes (SOD and CAT) in 21 children treated with VPA for one year and in 26 healthy children. We observed a significant increase of 10% in the levels of NO^•^ in children treated with VPA, compared with healthy children. There were no significant differences in the levels of lipid peroxidation and antioxidant enzymes.	[61]

—	GPX and MDA	G$\ddot{\text{u}}$neş et al., in 2009, analyzed the erythrocyte antioxidant status of 31 children with febrile seizures and 30 febrile children without seizures. The levels of lipid peroxidation were significantly higher, and the GPx and SOD levels were significantly lower in the group of children with febrile seizures compared with the group that did not present with seizures.	[62]

VPA, carbamazepine, and levetiracetam	8-OHG	Varoglu et al., in 2010, determined in 32 patients treated with VPA, 17 treated with carbamazepine, 8 with levetiracetam, and 11 with polytherapy that the levels of low-density lipoprotein (LDL) and 8-OHG were significantly higher in all patients, compared with the control group. Comparing the monotherapy versus the polytherapy, only the valproate + levetiracetam combination yielded a significant increase in 8-OHG.	[63]

OXC	NO^•^ and MDA	Arhan et al., in 2011, found a significant decrease in the serum levels of NO^•^ and lipid peroxidation in 16 children diagnosed with idiopathic epilepsy and treated for three months with OXC.	[64]

VPA	SOD, CAT, MPO, and MDA	Y.J. Zhang et al., in 2011, reported a significant decrease in the antioxidant activity of SOD and CAT. They also found a significant increase in the MPO activity and lipid peroxidation levels. This study was performed in 26 epileptic children treated for six and 12 months with VPA, compared with 30 healthy children.	[65]

## References

[1] Lara T (2002). Análisis Clínico-epidemiológico de la Epilepsia en la hospitalización psiquiátrica del Instituto Nacional de Neurología y Neurocirugía “Manuel Velasco Suárez” Una revisión de cuatro años. *Revista Argentina De Neurología, Psiquiatría Y Neurocirugía*.

[2] Engel J (1995). Concepts of epilepsy. *Epilepsia*.

[3] Lara TH, Ramírez RL (1993). Epidemiología de la Epilepsia en México. Un análisis interinstitucional de veinticinco años. *Revista Neurologia, Neurocirugia Psiquiatria*.

[4] Jacobs MP, Fischbach GD, Davis MR (2001). Future directions for epilepsy research. *Neurology*.

[5] Auvin S, Pineda E, Shin D, Gressens P, Mazarati A (2012). Novel animal models of pediatric epilepsy. *Neurotherapeutics*.

[6] ILAE, Commision on Classification and Terminology of the International League Against Epilepsy (1981). Proposal for revised clinical and electroencephalographic classification of epileptic seizures. From the Commission on Classification and Terminology of the International League Against Epilepsy. *Epilepsia*.

[7] ILAE (1989). Proposal for revised classification of epilepsies and epileptic syndromes. Commission on Classification and Terminology of the International League Against Epilepsy. *Epilepsia*.

[8] Reynolds EH (2002). Introduction: epilepsy in the world. *Epilepsia*.

[9] Sentíes-Madrid H Guías del Capítulo Mexicano de la Liga Internacional contra la Epilepsia. Antiepilépticos de primera generación contra antiepilépticos de segunda generación: énfasis en efectos adversos de la segunda generación.

[10] Shorvon SD (2009). Drug treatment of epilepsy in the century of the ILAE: the second 50 years, 1959–2009. *Epilepsia*.

[11] Löscher W, Schmidt D (2011). Modern antiepileptic drug development has failed to deliver: ways out of the current dilemma. *Epilepsia*.

[12] Loscher W (1993). In vivo administration of valproate reduces the nerve terminal (synaptosomal) activity of GABA aminotransferase in discrete brain areas of rats. *Neuroscience Letters*.

[13] Biggs CS, Pearce BR, Fowler LJ, Whitton PS (1992). The effect of sodium valproate on extracellular GABA and other amino acids in the rat ventral hippocampus: an in vivo microdialysis study. *Brain Research*.

[14] Rowley HL, Marsden CA, Martin KF (1995). Differential effects of phenytoin and sodium valproate on seizure-induced changes in *γ*-aminobutyric acid and glutamate release in vivo. *European Journal of Pharmacology*.

[15] Vriend JP, Alexiuk NAM (1996). Effects of valproate on amino acid and monoamine concentrations in striatum of audiogenic seizure-prone Balb/c mice. *Molecular and Chemical Neuropathology*.

[16] Bolanos JP, Medina JM (1993). Evidence of stimulation of the *γ*-aminobutyric acid shunt by valproate and E-Δ2-valproate in neonatal rat brain. *Molecular Pharmacology*.

[17] Loescher W (1981). Effect of inhibitors of GABA Aminotransferase on the metabolism of GABA in brain tissue and synaptosomal fractions. *Journal of Neurochemistry*.

[18] van der Laan JW, de Boer Th. DBT, Bruinvels J (1979). Di-n-propylacetate and GABA degradation. Preferential inhibition of succinic semialdehyde dehydrogenase and indirect inhibition of GABA-transaminase. *Journal of Neurochemistry*.

[19] Whittle SR, Turner AJ (1978). Effects of anticonvulsant sodium valproate on *γ*-aminobutyrate and aldehyde metabolism in ox brain. *Journal of Neurochemistry*.

[20] Collins RM, Zielke HR, Woody RC (1994). Valproate increases glutaminase and decreases glutamine synthetase activities in primary cultures of rat brain astrocytes. *Journal of Neurochemistry*.

[21] Cutrer FM, Limmroth V, Ayata G, Moskowitz MA (1995). Attenuation by valproate of c-fos immunoreactivity in trigeminal nucleus caudalis induced by intracisternal capsaicin. *British Journal of Pharmacology*.

[22] Zeise ML, Kasparow S, Zieglgansberger W (1991). Valproate suppresses N-methyl-D-aspartate-evoked transient depolarizations in the rat neocortex in vitro. *Brain Research*.

[23] Gean P-W, Huang C-C, Hung C-R, Tsai J-J (1994). Valproic acid suppresses the synaptic response mediated by the NMDA receptors in rat amygdalar slices. *Brain Research Bulletin*.

[24] Dreier JP, Heinemann U (1990). Late low magnesium-induced epileptiform activity in rat entorhinal cortex slices becomes insensitive to the anticonvulsant valproic acid. *Neuroscience Letters*.

[25] Buchhalter JR, Dichter MA (1986). Effects of valproic acid in cultured mammalian neurons. *Neurology*.

[26] Farrant M, Webster RA (1989). Neuronal activity, amino acid concentration and amino acid release in the substantia nigra of the rat after sodium valproate. *Brain Research*.

[27] Gaillard WD, Zeffiro T, Fazilat S, DeCarli C, Theodore WH (1996). Effect of valproate on cerebral metabolism and blood flow: an 18F-2-deoxyglusose and 15O water positron emission tomography study. *Epilepsia*.

[28] May TW, Korn-Merker E, Rambeck B (2003). Clinical pharmacokinetics of oxcarbazepine. *Clinical Pharmacokinetics*.

[29] Stefani A, Pisani A, de Murtas M, Mercuri NB, Marciani MG, Calabresi P (1995). Action of GP 47779, the active metabolite of oxcarbazepine, on the corticostriatal system. II. Modulation of high-voltage-activated calcium currents. *Epilepsia*.

[30] Waldmeier PC, Baumann PA, Wicki P, Feldtrauer J-J, Stierlin C, Schmutz M (1995). Similar potency of carbamazepine, oxcarbazepine, and lamotrigine in inhibiting the release of glutamate and other neurotransmitters. *Neurology*.

[31] DeLorenzo RJ, Sombati S, Coulter DA (2000). Effects of topiramate on sustained repetitive firing and spontaneous recurrent seizure discharges in cultured hippocampal neurons. *Epilepsia*.

[32] White HS, Brown SD, Woodhead JH, Skeen GA, Wolf HH (1997). Topiramate enhances GABA-mediated chloride flux and GABA-evoked chloride currents in murine brain neurons and increases seizure threshold. *Epilepsy Research*.

[33] White HS, Brown SD, Woodhead JH, Skeen GA, Wolf HH (2000). Topiramate modulates GABA-evoked currents in murine cortical neurons by a nonbenzodiazepine mechanism. *Epilepsia*.

[34] Valko M, Rhodes CJ, Moncol J, Izakovic M, Mazur M (2006). Free radicals, metals and antioxidants in oxidative stress-induced cancer. *Chemico-Biological Interactions*.

[35] Inoue M, Sato EF, Nishikawa M (2003). Mitochondrial generation of reactive oxygen species and its role in aerobic life. *Current Medicinal Chemistry*.

[36] Waldbaum S, Patel M (2010). Mitochondria, oxidative stress, and temporal lobe epilepsy. *Epilepsy Research*.

[37] Cardenas-Rodriguez N, Huerta-Gertrudis B, Rivera-Espinosa L (2013). Role of oxidative stress in refractory epilepsy: evidence in patients and experimental models. *International Journal of Molecular Sciences*.

[38] Ansari MA, Ahmad AS, Ahmad M (2004). Selenium protects cerebral ischemia in rat brain mitochondria. *Biological Trace Element Research*.

[39] Bondy SC (1995). The relation of oxidative stress and hyperexcitation to neurological disease. *Proceedings of the Society for Experimental Biology and Medicine*.

[40] Dalton T (1995). Temporalspatial patterns of expression of metallothionein-I and -III and other stress related genes in rat brain after kainic acid-induced seizures. *Neurochemistry International*.

[41] Patel M, Day BJ, Crapo JD, Fridovich I, McNamara JO (1996). Requirement for superoxide in excitotoxic cell death. *Neuron*.

[42] Uma Devi P, Kolappa Pillai K, Vohora D (2006). Modulation of pentylenetetrazole-induced seizures and oxidative stress parameters by sodium valproate in the absence and presence of N-acetylcysteine. *Fundamental and Clinical Pharmacology*.

[43] Armağan A, Kutluhan S, Yilmaz M (2008). Topiramate and vitamin E modulate antioxidant enzyme activities, nitric oxide and lipid peroxidation levels in pentylenetetrazol-induced nephrotoxicity in rats. *Basic and Clinical Pharmacology and Toxicology*.

[44] Frantseva MV, Perez Velazquez JL, Tsoraklidis G (2000). Oxidative stress is involved in seizure-induced neurodegeneration in the kindling model of epilepsy. *Neuroscience*.

[45] Schweizer U, Bräuer AU, Köhrle J, Nitsch R, Savaskan NE (2004). Selenium and brain function: a poorly recognized liaison. *Brain Research Reviews*.

[46] Kovács R, Schuchmann S, Gabriel S, Kann O, Kardos J, Heinemann U (2002). Free radical-mediated cell damage after experimental status epilepticus in hippocampal slice cultures. *Journal of Neurophysiology*.

[47] White HS, Smith MD, Wilcox KS (2007). Mechanisms of action of antiepileptic drugs. *International Review of Neurobiology*.

[48] Singh P, Mann KA, Mangat HK, Kaur G (2003). Prolonged glutamate excitotoxicity: effects on mitochondrial antioxidants and antioxidant enzymes. *Molecular and Cellular Biochemistry*.

[49] Granger DN (1988). Role of xanthine oxidase and granulocytes in ischemia-reperfusion injury. *American Journal of Physiology—Heart and Circulatory Physiology*.

[50] Hensley K, Maidt ML, Pye QN (1997). Quantitation of protein-bound 3-nitrotyrosine and 3,4-dihydroxyphenylalanine by high-performance liquid chromatography with electrochemical array detection. *Analytical Biochemistry*.

[51] Kurekci AE, Alpay F, Tanindi S (1995). Plasma trace element, plasma glutathione peroxidase, and superoxide dismutase levels in epileptic children receiving antiepileptic drug therapy. *Epilepsia*.

[52] Cengiz M, Yüksel A, Seven M (2000). The effects of carbamazepine and valproic acid on the erythrocyte glutathione, glutathione peroxidase, superoxide dismutase and serum lipid peroxidation in epileptic children. *Pharmacological Research*.

[53] Yüksel A, Cengiz M, Seven M, Ulutin T (2000). Erythrocyte glutathione, glutathione peroxidase, superoxide dismutase and serum lipid peroxidation in epileptic children with valproate and carbamazepine monotherapy. *Journal of Basic and Clinical Physiology and Pharmacology*.

[54] Yüksel A, Cengiz M, Seven M, Ulutin T (2001). Changes in the antioxidant system in epileptic children receiving antiepileptic drugs: two-year prospective studies. *Journal of Child Neurology*.

[55] Verrotti A, Basciani F, Trotta D, Pomilio MP, Morgese G, Chiarelli F (2002). Serum copper, zinc, selenium, glutathione peroxidase and superoxide dismutase levels in epileptic children before and after 1 year of sodium valproate and carbamazepine therapy. *Epilepsy Research*.

[56] Sołowiej E, Sobaniec W (2003). The effect of antiepileptic drug therapy on antioxidant enzyme activity and serum lipid peroxidation in young patients with epilepsy. *Neurologia i Neurochirurgia Polska*.

[57] Hamed SA, Abdellah MM, El-Melegy N (2004). Blood levels of trace elements, electrolytes, and oxidative stress/antioxidant systems in epileptic patients. *Journal of Pharmacological Sciences*.

[58] Bolayir E, Celik K, Tas A, Topaktas S, Bakir S (2004). The effects of oxcarbazepine on oxidative stress in epileptic patients. *Methods and Findings in Experimental and Clinical Pharmacology*.

[59] Martínez-Ballesteros C, Pita-Calandre E, Sánchez-González Y, Rodríguez-López CM, Agil A (2004). Lipid peroxidation in adult epileptic patients treated with valproic acid. *Revista de Neurologia*.

[60] Sobaniec W, Solowiej E, Kulak W, Bockowski L, Smigielska-Kuzia J, Artemowicz B (2006). Evaluation of the influence of antiepileptic therapy on antioxidant enzyme activity and lipid peroxidation in erythrocytes of children with epilepsy. *Journal of Child Neurology*.

[61] Peker E, Oktar S, Ari M (2009). Nitric oxide, lipid peroxidation, and antioxidant enzyme levels in epileptic children using valproic acid. *Brain Research*.

[62] Güneş S, Dirik E, Yiş U (2009). Oxidant status in children after febrile seizures. *Pediatric Neurology*.

[63] Varoglu AO, Yildirim A, Aygul R, Gundogdu OL, Sahin YN (2010). Effects of valproate, carbamazepine, and levetiracetam on the antioxidant and oxidant systems in epileptic patients and their clinical importance. *Clinical Neuropharmacology*.

[64] Arhan E, Serdaroglu A, Ozturk B (2011). Effects of epilepsy and antiepileptic drugs on nitric oxide, lipid peroxidation and xanthine oxidase system in children with idiopathic epilepsy. *Seizure*.

[65] Zhang YJ, Zhang M, Wang XC (2011). Effects of sodium valproate on neutrophils' oxidative metabolism and oxidant status in children with idiopathic epilepsy. *Chinese Journal of Pediatrics*.

[66] Chaudhary S, Parvez S (2012). An in vitro approach to assess the neurotoxicity of valproic acid-induced oxidative stress in cerebellum and cerebral cortex of young rats. *Neuroscience*.

[67] Yiş U, Seçkin E, Kurul SH, Kuralay F, Dirik E (2009). Effects of epilepsy and valproic acid on oxidant status in children with idiopathic epilepsy. *Epilepsy Research*.

[68] Aycicek A, Iscan A (2007). The effects of carbamazepine, valproic acid and phenobarbital on the oxidative and antioxidative balance in epileptic children. *European Neurology*.

[69] Seçkin Ş, Başaran-Küçükgergin C, Uysal M (1999). Effect of acute and chronic administration of sodium valproate on lipid peroxidation and antioxidant system in rat liver. *Pharmacology and Toxicology*.

[70] Cardile V, Pavone A, Renis M, Maci T, Perciavalle V (2001). Effects of gabapentin and topiramate in primary rat astrocyte cultures. *NeuroReport*.

[71] Pavone A, Cardile V (2003). An in vitro study of new antiepileptic drugs and astrocytes. *Epilepsia*.

[72] Agarwal NB, Agarwal NK, Mediratta PK, Sharma KK (2011). Effect of lamotrigine, oxcarbazepine and topiramate on cognitive functions and oxidative stress in PTZ-kindled mice. *Seizure*.

[73] Muriach M, López-Pedrajas R, Barcia JM, Sanchez-Villarejo MV, Almansa I, Romero FJ (2010). Cocaine causes memory and learning impairments in rats: involvement of nuclear factor kappa B and oxidative stress, and prevention by topiramate. *Journal of Neurochemistry*.

[74] Kutluhan S, Naziroğlu M, Çelik Ö, Yilmaz M (2009). Effects of selenium and topiramate on lipid peroxidation and antioxidant vitamin levels in blood of pentylentetrazol-induced epileptic rats. *Biological Trace Element Research*.

[75] Naziroğlu M, Uğuz AC, Gokçimen A (2008). Tenoxicam modulates antioxidant redox system and lipid peroxidation in rat brain. *Neurochemical Research*.

[76] Kubera M, Budziszewska B, Jaworska-Feil L (2004). Effect of topiramate on the kainate-induced status epilepticus, lipid peroxidation and immunoreactivity of rats. *Polish Journal of Pharmacology*.

[77] Cardenas-Rodriguez N, Coballase-Urrutia E, Huerta-Gertrudis B (2013). Antioxidant activity of topiramate: an antiepileptic agent. *Neurological Sciences*.

